# UV-Photofunctionalization of Titanium Promotes Mechanical Anchorage in A Rat Osteoporosis Model

**DOI:** 10.3390/ijms21041235

**Published:** 2020-02-12

**Authors:** Takashi Taniyama, Juri Saruta, Naser Mohammadzadeh Rezaei, Kourosh Nakhaei, Amirreza Ghassemi, Makoto Hirota, Takahisa Okubo, Takayuki Ikeda, Yoshihiko Sugita, Masakazu Hasegawa, Takahiro Ogawa

**Affiliations:** 1 Weintraub Center for Reconstructive Biotechnology, Division of Advanced Prosthodontics, UCLA School of Dentistry, Los Angeles, CA 90095-1668, USA; taniyama.ortho@tmd.ac.jp (T.T.); naser.m.rezaei@gmail.com (N.M.R.); kourosh1988@ucla.edu (K.N.); a.r_ghassemi_dent@yahoo.com (A.G.); mhirota@yokohama-cu.ac.jp (M.H.); okubotakahisa@gmail.com (T.O.); ikeda.takayuki@nihon-u.ac.jp (T.I.); yosshii@dpc.agu.ac.jp (Y.S.); masa.hasegawa0202@gmail.com (M.H.); togawa@dentistry.ucla.edu (T.O.); 2 Department of Orthopedic Surgery, Yokohama City Minato Red Cross Hospital, 3-12-1 Shinyamashita, Yokohama 231-8682, Kanagawa, Japan; 3 Department of Oral Science, Graduate School of Dentistry, Kanagawa Dental University, 82 Inaoka, Yokosuka 238-8580, Kanagawa, Japan; 4 Department of Oral and Maxillofacial Surgery/Orthodontics, Yokohama City University Medical Center, 4-57 Urafune-cho, Yokohama 232-0024, Kanagawa, Japan

**Keywords:** titanium, osteoblast, osteoporosis, UV light treatment, bone regeneration, ovariectomy, bone integration, mechanical anchorage, dental implants, orthopedic implants

## Abstract

Effects of UV-photofunctionalization on bone-to-titanium integration under challenging systemic conditions remain unclear. We examined the behavior and response of osteoblasts from sham-operated and ovariectomized (OVX) rats on titanium surfaces with or without UV light pre-treatment and the strength of bone-implant integration. Osteoblasts from OVX rats showed significantly lower alkaline phosphatase, osteogenic gene expression, and mineralization activities than those from sham rats. Bone density variables in the spine were consistently lower in OVX rats. UV-treated titanium was superhydrophilic and the contact angle of ddH_2_O was ≤5°. Titanium without UV treatment was hydrophobic with a contact angle of ≥80°. Initial attachment to titanium, proliferation, alkaline phosphatase activity, and gene expression were significantly increased on UV-treated titanium compared to that on control titanium in osteoblasts from sham and OVX rats. Osteoblastic functions compromised by OVX were elevated to levels equivalent to or higher than those of sham-operated osteoblasts following culture on UV-treated titanium. The strength of in vivo bone-implant integration for UV-treated titanium was 80% higher than that of control titanium in OVX rats and even higher than that of control implants in sham-operated rats. Thus, UV-photofunctionalization effectively enhanced bone-implant integration in OVX rats to overcome post-menopausal osteoporosis-like conditions.

## 1. Introduction

Osteoporosis is a major public health care problem that is increasing with the aging population [1]. This progressive bone disorder is characterized by low bone mass, microstructure, and bone fragility, resulting in an increased risk of fracture [2]. Currently, 10.3 million people have osteoporosis and another 43.4 million older adults are at risk of osteoporosis or osteopenia in the United States. Chin et al. reported that among patients older than 50 years, the incidence of osteoporosis was 14.5% in men and 51.3% in women [3].

Additionally, osteoporosis is an important disease state in orthopedic and dental implant surgery. Complications associated with spinal implant surgery in patients with osteoporosis are common [4,5]. Particularly, vertebral fractures and loosening of pedicle screws after implant surgery are the most frequent complications [6]. In dental implant surgery, osteoporosis is considered as a risk factor for implant surgery. Many clinical studies or systematic reviews have suggested that osteoporotic subjects have higher rates of implant failure [7,8,9]. To resolve these challenges following implant surgery in patients with osteoporosis, implants that are more osseointegrative and biocompatible as well as improved titanium devices are necessary to improve clinical outcomes.

Ultraviolet (UV)-mediated photofunctionalization is a method of surface modification for titanium to increase its biologic capacity; this approach is characterized by remarkable efficacy, unique mechanisms, and simple delivery [10,11]. Photofunctionalization results in nearly 100% bone-to-implant contact and three-fold greater mechanical anchorage strength compared to the untreated titanium surfaces in animal models [10,12]. These effects occur because of the removal of carbon compounds (such as hydrocarbons) accumulated on titanium surfaces [13,14,15]. Although many patients who undergo orthopedic or dental implant surgery have osteoporosis and are elderly, the effects of UV photofunctionalization under osteoporotic conditions have not been determined. During the onset of osteoporosis, the primary effect on the compromised osteogenesis is through osteoblasts. Because of menopause, estrogen production is decreased, which impairs the ability of osteoblasts to synthesize bone matrix, which may result in less bone-implant contact with lower torque resistance [16]. Based on previous studies, there are currently at least three key mechanisms by which estrogen deficiency may lead to a relative deficit in bone formation through direct effects on osteoblasts: increased apoptosis, increased oxidative stress, and increased nuclear factor-kappa B activity [17]. Ovariectomized (OVX) animal models are considered as appropriate for mimicking the conditions in post-menopausal women and have been widely used to evaluate potential therapeutics for preventing or treating osteoporosis [18]. However, how osteoblasts extracted from rats in these osteoporosis models behave on titanium surfaces is unclear. This study was conducted to examine the effects of UV-photofunctionalization of titanium on various in vitro behaviors and functions of osteoblasts on a titanium substrate and in vivo potential of bone-titanium integration in an osteoporosis rat model.

## 2. Results

### 2.1. Generation of Hydrophilic Surface on UV-Treated Titanium

**Figure 1:**
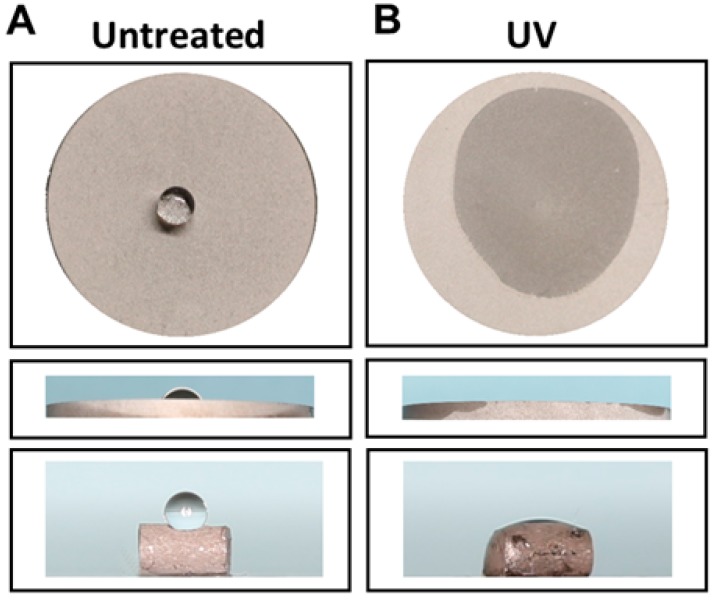
Converted hydrophilicity of acid-etched titanium disk and implant surface. (**A**) Hydrophobic surface of untreated acid-etched disk and implant. (**B**) Hydrophilic surface obtained by photofunctionalization (UV-treated), on which water immediately spread across the surface.

There was a marked difference in wettability before and after UV-photofunctionalization (Figure 1A,B). A 10-µL double-distilled H_2_O (ddH_2_O) droplet placed on regular (untreated) acid-etched titanium disks and implants remained hemispheric, indicating that the surfaces were hydrophobic, whereas water placed onto UV-treated disks and implants immediately spread over nearly the entire area of the surfaces, indicating that the surfaces were superhydrophilic. The ddH_2_O contact angle was 80° or higher on untreated acid-etched titanium surfaces and 5° or less on UV-treated surfaces.

### 2.2. Osteoblast Attachment to Titanium with or Without UV Treatment

**Figure 2:**
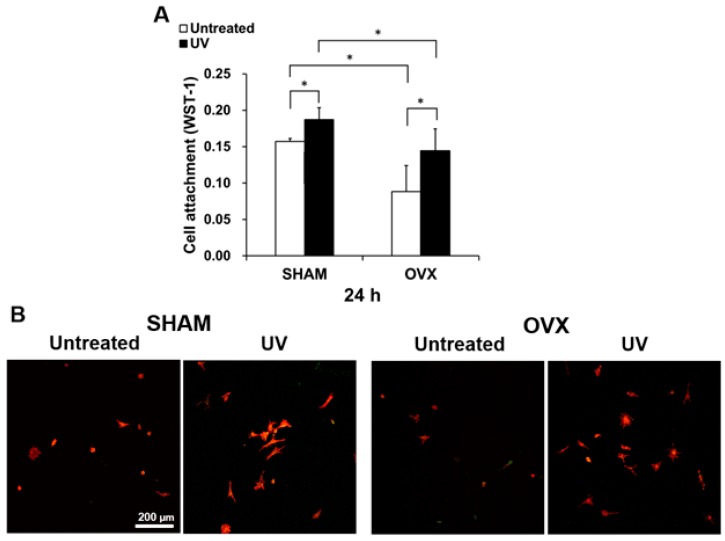
Cell attachment of osteoblasts from sham and OVX groups on acid-etched titanium surfaces with or without UV. (**A**) Number of attached cells after 24 h-incubations evaluated by WST-1 assay. (**B**) Initial spread of osteoblasts at 24 h after seeding on titanium surface. Representative confocal microscopy images of cells stained with rhodamine phalloidin for actin filaments (red) and anti-vinculin for vinculin (green). Each value represents the mean ± standard deviation from triplicate experiments (*n* = 3). * *p* < 0.05, significant between untreated and photofunctionalized surfaces. Scale bar = 200 µm.

The number of osteoblasts from sham and OVX rats attached to the titanium disks was evaluated by WST-1 assay at 24 h after seeding. Regardless of the UV treatment of titanium, a larger number of osteoblasts derived from sham-operated animals than from OVX animals were attached to the titanium (Figure 2A). Specifically, half the number of cells were attached to untreated titanium disks in the OVX group compared to in the sham-operated group. Comparison of the untreated and UV-treated titanium disks showed that more osteoblasts attached to UV-treated disks than to untreated disks. Notably, the number of osteoblasts from the OVX group attached to UV-treated titanium was comparable to that attached to untreated titanium in the sham-operated group. These quantitative data were supported by the low-magnification confocal microscopic images showing an increased number of cells attached to UV-treated titanium both in the sham-operated and OVX groups (Figure 2B).

### 2.3. Spreading Behavior of Osteoblasts on Titanium with or Without UV Treatment

**Figure 3:**
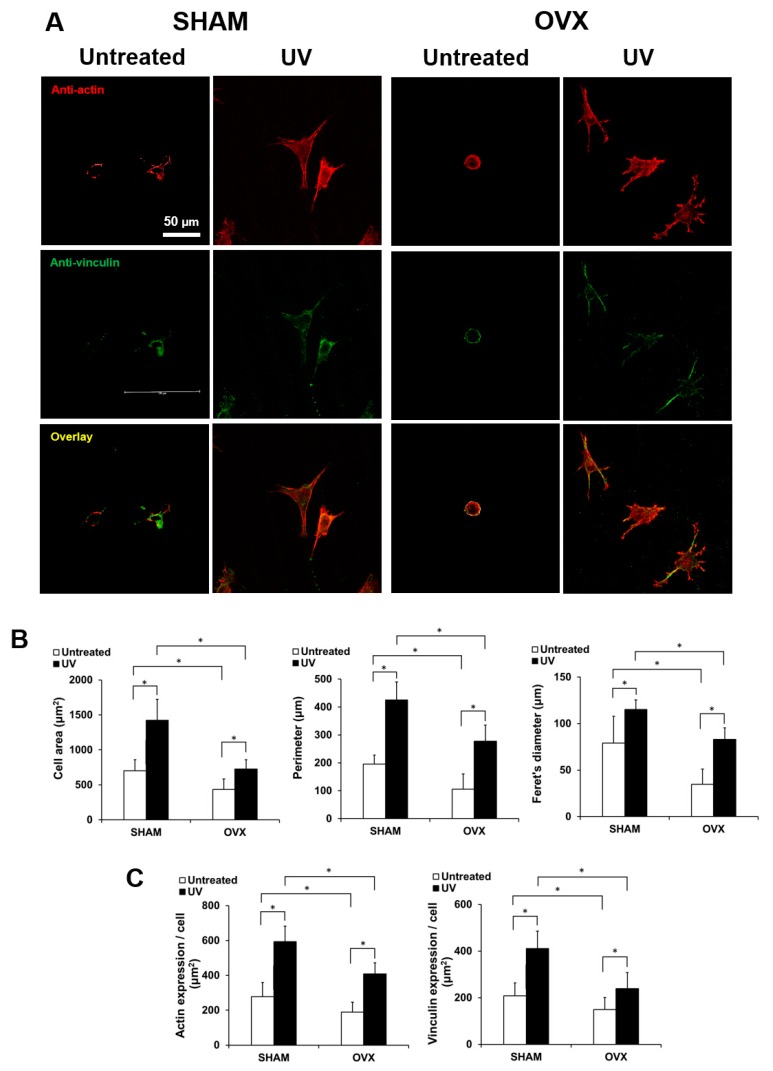
Representative confocal microscopy images of the spreading behavior of osteoblasts from sham and OVX groups at 24 h after seeding on untreated and photofunctionalized titanium surfaces. (**A**) Confocal microscopic images of osteoblast following immunochemical staining for cytoskeletal actin and adhesion protein vinculin are shown. Scale bar = 50 µm. (**B**) Histograms for cytomorphometric parameters measured from the images. (**C**) The expression levels of actin and vinculin were semiquantified using the confocal microscopy images. Data are mean ± standard deviation from triplicate experiments (*n* = 3). * *p* < 0.05, significant between untreated and photofunctionalized surfaces.

After 24 h of incubation, the cells were dual-stained and examined by confocal laser microcopy to determine their attachment and spreading behaviors (Figure 3A). Cells appeared larger on UV-treated titanium than on untreated titanium both in the sham-operated and OVX groups. In addition to their enlarged size, cells on UV-treated titanium showed more developed lamellipodia- and filopodia-like cytoplasmic projections (Figure 3A,B). Further, the expression of cytoskeletal actin and vinculin, a focal adhesion protein, was more intense and extensive in cells on UV-treated titanium (Figure 3C). There was no observable difference between the sham-operated and OVX groups.

### 2.4. Osteoblast Proliferation on Titanium with or Without UV Treatment

**Figure 4:**
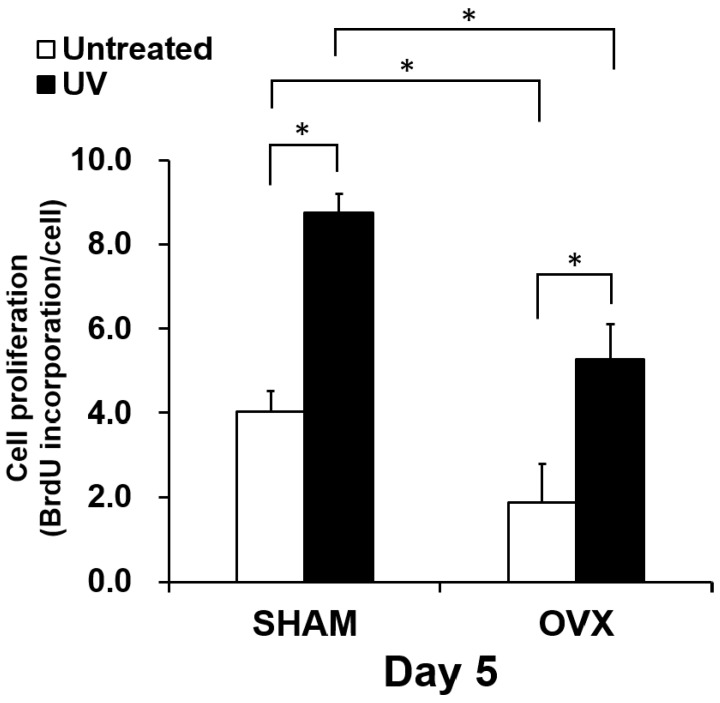
Proliferative activity of osteoblasts from sham and OVX groups on untreated and photofunctionalized titanium surfaces. 5-Bromo-2′ deoxyuridine (BrdU) incorporation into DNA during mitosis standardized by cell number. BrdU incorporation into DNA measured on day 5. Each value represents the mean ± standard deviation from triplicate experiments (*n* = 3). * *p* < 0.05, significant difference between untreated and photofunctionalized surfaces.

The proliferative activity of osteoblasts was evaluated by measuring BrdU incorporation into DNA on day 5 (Figure 4). Similar to the results of the number of attached cells, proliferation activity was significantly reduced in the OVX group compared to in the sham group. Cells in the OVX group showed approximately a half the activity of cells in the sham-operated group. There was more contract between the untreated and UV-treated titanium disks. Cell proliferation was increased on UV-treated titanium compared to on untreated titanium by 120% and 250%, respectively, in the Sham and OVX groups. The proliferation of OVX-group osteoblasts on UV-treated titanium was equivalent to that of sham-group osteoblasts on untreated titanium.

### 2.5. Osteoblastic Differentiation on Titanium with or Without UV Treatment

**Figure 5:**
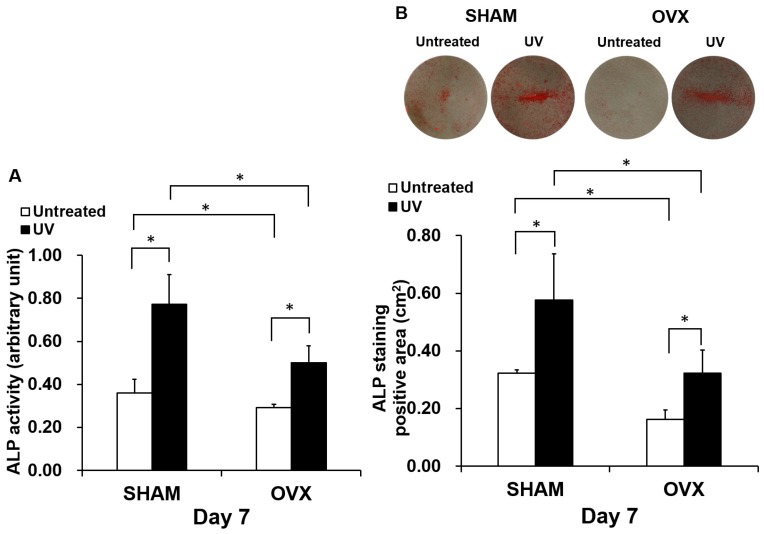
UV light-enhanced alkaline phosphatase (ALP) activity, an early- to middle-stage marker of osteoblasts, in the OVX and sham groups. (**A**) Colorimetrically quantified ALP activity standardized per cell at day 7 is shown. (**B**) ALP activity measured as the ALP-positive (red) area at day 7. Representative images of the stained culture are presented on the top. The ALP-positive area measured from the images is presented at the bottom histogram in square centimeters. Each value represents the mean ± standard deviation of triplicate experiments (*n* = 3). * *p* < 0.05, significant difference between untreated and UV light-treated surfaces.

**Figure 6:**
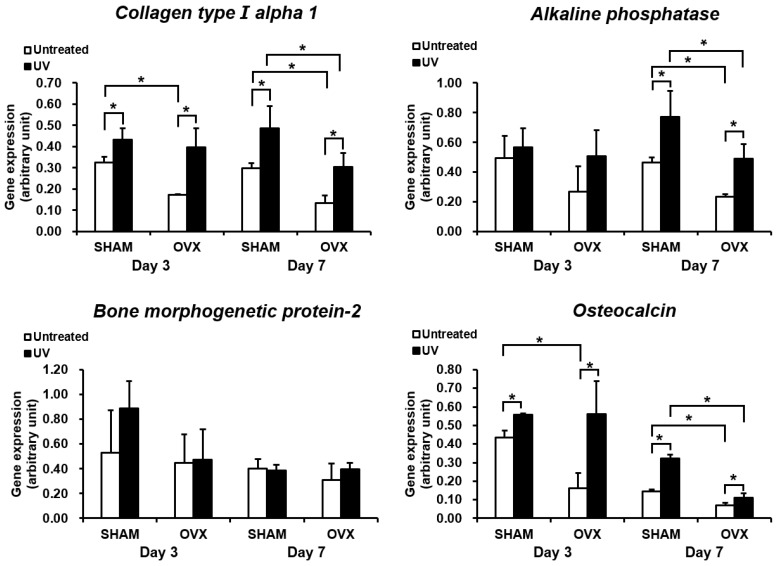
Gene expression levels of selected markers quantified by real-time qPCR. The osteogenic markers collagen type I alpha 1, alkaline phosphatase, bone morphogenetic protein-2, and osteocalcin were analyzed. Total RNA was isolated at 3 and 7 days from osteoblastic cell cultures on acid-etched titanium surfaces with or without UV-treatment. Relative expression levels (2^−ΔΔCt^ values) of the genes of interest were normalized to that of the housekeeping gene *Gapdh*. Each value represents the mean ± standard deviation of triplicate experiments (*n* = 3). * *p* < 0.05, significant difference between untreated and UV light-treated surfaces.

Osteoblastic differentiation was evaluated by measuring ALP activity and the expression of bone-related genes. As observed for the number of attached cells and proliferation, ALP activity was significantly reduced in the OVX group (Figure 5A). However, ALP activity was substantially increased by culturing the cells on UV-treated titanium both in the sham and OVX groups. Again, OVX-group cells on UV-treated titanium showed similar ALP activity as sham group cells on untreated titanium. The images from ALP staining (top panels in Figure 5B) confirmed the results of ALP chemical quantification. We next examined the expression of bone-related genes on days 3 and 7 (Figure 6). The level of gene expression was significantly lower in the OVX group than in the sham-operated group. This trend was more typical on untreated titanium disks in the OVX group. Cells cultured on UV-treated titanium showed significantly upregulated expression of all genes tested except for *BMP-2*. Because of this upregulation, OVX-group cells on UV-treated titanium showed gene expression equivalent to or even higher than that of sham-group cells on untreated titanium for most genes tested. The results were consistent between days 3 and 7.

### 2.6. In Vivo Mechanical Anchorage of Titanium with or Without UV Treatment

**Figure 7:**
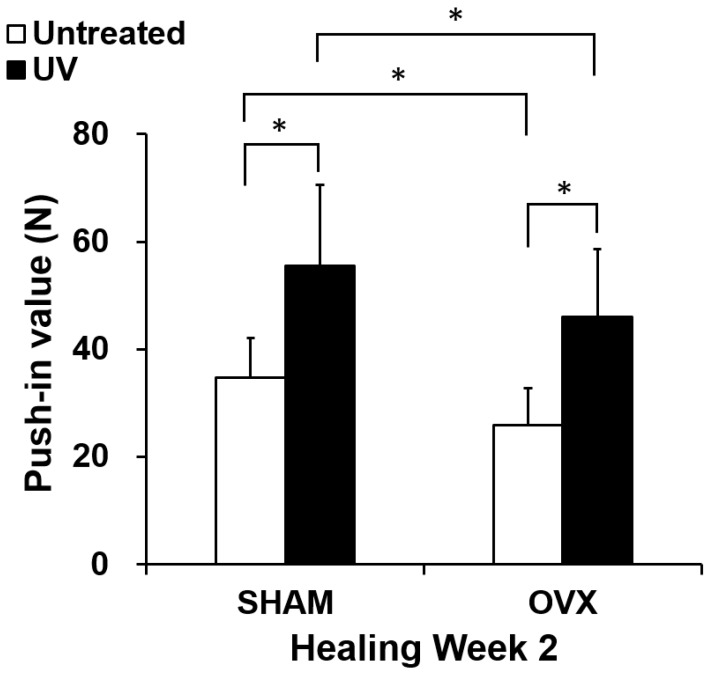
Strength of implant anchorage in bone evaluated by the biomechanical push-in test in OVX and sham rat femur models. Each bar represents the mean ± standard deviation of the sham-untreated, sham-UV, OVX-untreated, and OVX-UV implant groups (each *n* = 8; healing time 2 weeks), * *p* < 0.05, significant difference between untreated and UV light-treated surfaces.

The strength of bone and titanium integration was assessed by the biomechanical push-in test at week 2 of healing (Figure 7). Using untreated titanium implants, the push-in value was significantly lower in the OVX group than in the sham group. The use of UV-treated implants increased the push-in value in both the sham and OVX groups by 50–70%. The push-in value for UV-treated implants in the OVX group was equivalent to that for untreated implants in sham-operated animals.

## 3. Discussion

We demonstrated that osteoblasts extracted from OVX rats showed poor proliferation and differentiation capacity compared to those from sham rats. However, UV-treatment compensated for the poor osteoblastic proliferation and differentiation in OVX rats. Additionally, the push-in value for OVX rats obtained by UV-treated implants was higher than that for sham rats with untreated implants at week 2.

Previous animal experiments using an ovariectomy model of osteoporosis induction with implants inserted in rats showed that estrogen deficiency results in lower bone-to-implant contact, bone/implant interface biomechanical competence, and bone density on cancellous bone [19,20,21]. Giro et al. performed implant surgery in OVX rats and found that ovariectomy resulted in a reduction in peri-implant bone mineral density [21]. Some prior studies have shown conflicting findings [22,23]; however, Goergen et al. showed that the osteoblastic differentiation potential of bone marrow mesenchymal stem cells (BMMSCs) was increased in both an OVX + diet group and OVX + steroid group compared to in sham rats, and BMMSCs from OVX + diet rats exhibited higher matrix mineralization and unregulated mRNA expression of osteoblastic markers [22]. Rodríguez et al. found that BMMSCs derived from women with menopausal osteoporosis aged 65–75 years had a higher adipogenic capacity but had 50% lower type-I collagen synthesis and 60% lower transforming growth factor β expression [23]. These observations do not completely agree with our results, which may be because of variability in the animal models used and individual differences.

In accordance with previous studies revealing the presence of estrogen receptors in osteoclasts, a recent study demonstrated that osteoclasts are direct targets of estrogen, which is involved in regulating osteoclast apoptosis [24]. Estrogen also appears to regulate osteoclast formation and activity indirectly. Estrogen suppresses RANKL production by osteoblasts, T cells, and B cells [25]. Moreover, estrogen has also been shown to modulate the production of numerous bone-resorbing cytokines, including interleukin-1, interleukin-6, tumor necrosis factor-α [26,27,28,29]. Estrogen also affects osteoblasts by inhibiting osteoblast apoptosis and increasing osteoblast lifespan, thereby increasing the functional capacity of each cell [30]. Moreover, products of oxidative stress, including reactive oxygen species, attenuate osteoblastogenesis and decrease osteoblast/osteocyte lifespan. Interestingly the effects of aging on oxidative stress are recapitulated by the loss of estrogen; moreover, the effects of estrogen deficiency on bone are reversed by antioxidants [31]. These findings suggest that the lower ALP, osteogenic gene expression, and mineralization observed in osteoblast derived from OVX rats compared to sham rats may have been caused by estrogen deficiency through increased osteoblast apoptosis and increased oxidative stress.

**Figure 8:**
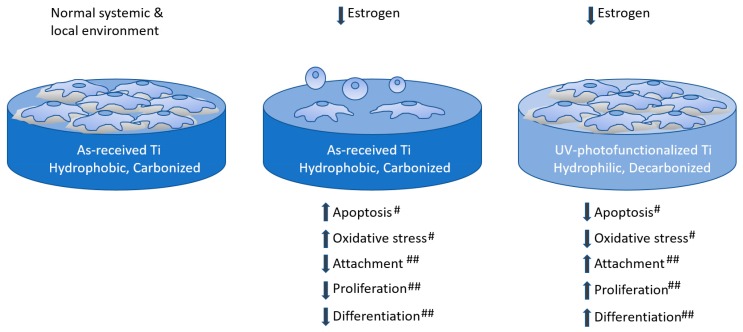
Scheme illustrating the proposed effect underlying the enhancement of attachment, proliferation, and differentiation of osteoblasts by UV-photofunctionalization in osteoporosis model. “#” and “##” indicates the associated strength.

UV light treatment has been reported to be an effective tool for cleaning accumulated organic contamination on medical devices. UV-treatment can remove hydrocarbon species from the surface of titanium and enhance bone conductivity [32,33,34,35,36]. Furthermore, photofunctionalization has yielded positive results in clinical studies [37,38]. In the present study, we demonstrated that the value of osteoblast proliferation increases and that of osteoblastic differentiation, as shown by the results of ALP activity and gene expression, is upregulated. We previously demonstrated that the intracellular production of reactive oxygen species, which induce apoptosis and are associated with osteoblast apoptosis in biomaterials [39,40], is significantly reduced by UV pretreatment of titanium [41]. Therefore, UV treatment appeared to compensate for the effect of increased osteoblast apoptosis and intracellular oxidative stress caused by estrogen deficiency; thus, photofunctionalization enhanced osteoblastic attachment on the titanium surface and cell proliferation without sacrificing differentiation in the ovariectomized osteoporosis model (Figure 8). This biological advantage enabled higher in vivo mechanical anchorage with the UV-treated titanium implant, which agrees with our previous reports [10,42,43,44].

Estrogen plays an important role in the maintenance of stable bone mass during adult life. In the present study, we used OVX and sham rats, the most commonly used experimental and control animal models for post-menopausal osteoporosis [45]. The duration from ovariectomization to sacrifice or isolation and culture of osteoblasts was 3 months. Bone turnover increase after ovariectomization, with the maximum increase occurring during the first month and continuing to approximately 100 days post-surgery [46]. Ocarino et al. demonstrated that 3 months after ovariectomy, all rats showed significantly reduced trabecular bone mass in the whole skeleton compared to in the sham group [47]. Indeed, our micro-CT based morphometric study showed that osteoporotic changes in the lumbar vertebra of OVX rats were observed 8 weeks after ovariectomy, which is consistent with these reports.

One limitation of this study is that we used an OVX model. A high turnover osteoporosis model in OVX animals appropriately reflects post-menopausal osteoporosis. However, low turnover osteoporosis as observed in elderly patients may differ in bone metabolism from that in the OVX animal model. Therefore, the OVX model may not precisely reflect the conditions of all elderly patients.

## 4. Materials and Methods

### 4.1. Titanium Samples Preparation

Disks (diameter, 20 mm; thickness, 1 mm) and cylindrical implants (diameter, 1 mm; length, 2 mm) were prepared from commercially pure titanium (grade 2) (Figure 1). Both titanium surface types were treated by regular acid-etching with 67% (*w*/*w*) sulfuric acid (Sigma-Aldrich, St. Louis, MO, USA) at 120 °C for 75 s. The surfaces were cleaned with 70% alcohol, sterilized by autoclaving, placed in a sealed container, and stored in a dark room (temperature, 23 °C; humidity, 60%) for 4 weeks until surgery or use in cell culture. Unthreaded cylindrical implants were used for the biomechanical implant push-in test, which measures the shear strength of the implant-bone integrated interface. For the UV treatment group, the titanium implants were photofunctionalized by treatment with UV light for 12 min using a photo device (Thera Beam^®^ SuperOsseo, Ushio, Inc., Tokyo, Japan) immediately before implantation or use in cell culture. For the non-treated group, we used the titanium implants without modification.

### 4.2. Animal Experiments

Female Sprague-Dawley (SD) rats (8-weeks-old) were purchased from Charles River Laboratories (Wilmington, MA, USA). The rats were housed in groups of two per cage and were maintained under pathogen-free, temperature- and humidity-controlled conditions (22 ± 3 °C, 55 ± 2%) under ambient conditions with 12-h dark and light cycles (lights on at 07:00 h). Animals were fed standard feed, and food and water were not limited. All experiments were performed following protocols approved by The Chancellor’s Animal Research Committee at the University of California at Los Angeles (ARC #2005-175-41E, approved on 30 January 2018), and according to the PHS Policy for the Humane Care and Use of Laboratory Animals and the UCLA Animal Care and Use Training Manual guidelines. SD rats (8 weeks of age) were randomly divided into two groups: one group of rats was sham-operated, and another group of rats underwent ovariectomy. OVX rats and sham rats were provided by Charles River Laboratories, and the OVX group underwent bilateral OVX using a sterile technique as described previously [48]. Rats in both groups were fed for 4 weeks after surgery.

### 4.3. Osteoblastic Cell Culture

At 4 weeks after sham or OVX surgery, bone marrow-derived osteoblasts were isolated from the femur of 12-week-old female SD rats. As previously described [49], extracted cells were cultured and the cells were incubated in a humidified atmosphere of 95% air and 5% CO_2_ at 37 °C. At 80% confluency, the cells were detached using 0.25% trypsin-1 mM EDTA-4Na and seeded onto titanium disks placed in a 12-well culture dish at a density of 3 × 10^4^ cells/cm^2^. The culture medium was renewed every 3 days.

### 4.4. Cell Attachment and Proliferation Assays

Initial attachment of cells was evaluated by measuring the number of cells attached to the titanium disks after 24 h of incubation in a WST-1-based colorimetric assay (WST-1, Roche Applied Science, Mannheim, Germany). As previously described [50], the amount of formazan product was measured with a multi-detection microplate reader, Synergy^TM^ HT (BioTek Instruments, Winooski, VT, USA), at a wavelength of 450 nm. Additionally, the proliferative activity of the cells was measured by BrdU incorporation during DNA synthesis on day 5 of culture [51]. Absorbance at 370 nm was measured with a Synergy^TM^ HT.

### 4.5. Alkaline Phosphatase (ALP) Activity

The ALP activity of cultured osteoblasts was examined in a colorimetry- and culture area-based assay. For colorimetry and culture area-based quantification, the culture well incubated after 7 days was rinsed twice with distilled water and 250 µL of *p*-nitrophenylphosphate (LabAssay ALP, Wako Pure Chemicals, Osaka, Japan), as previously described [52]. Absorbance at 405 nm was measured with a Synergy^TM^ HT. The ALP-positive area on the stained images was calculated with image analysis software (ImageJ, NIH, Bethesda, MD, USA).

### 4.6. Mineralization Staining

Von Kossa staining was performed to visualize the mineralized nodules of osteoblasts at 14 days. The cultures were fixed and incubated with 5% silver nitrate under UV light for 30 min, as previously described [52]. The cultures were washed with ddH_2_O and incubated with 5% sodium thiosulfate solution for 2–5 min. The Von Kossa-positive area on the stained images was calculated using ImageJ software.

### 4.7. Morphology and Morphometry of Osteoblastic Cells

Spreading behavior and cytoskeletal arrangement of osteoblasts seeded onto the titanium surfaces were examined by confocal laser scanning microscopy (TCS SP5, Leica, Wetzlar, Germany), as previously described [50]. After 24 h of culture, osteoblasts were fixed and stained with fluorescent rhodamine phalloidin dye (actin filament, red color; R415, Molecular Probes, Eugene, OR, USA) and vinculin (green color; ab11194, Abcam, Cambridge, UK).

### 4.8. Gene Expression Analysis (Quantitative Real-Time PCR)

Total RNA was extracted from the cells on days 3 and 7 using Trizol reagent (Life Technologies, Carlsbad, CA, USA) and purified using the Direct-zol^TM^ RNA MiniPrep kit (Zymo Research, Irvine, CA, USA) according to the manufacturer’s protocol [53]. Quantitative real-time PCR was performed in triplicate for each sample with an LC480 SYBR Green I master (Roche) using universal cycling conditions [54]. A total of 55 cycles was performed, and the second derivative Cq value determination method was used to compare fold-differences in expression. For PCR amplification, the target-specific PCR primers for collagen type I alpha 1 chain (*Col1a1*), alkaline phosphatase (*Alp*), bone morphogenetic protein-2 (*Bmp-2*), and osteocalcin (*Ocn*) were used. Glyceraldehyde-3-phosphate dehydrogenase (*Gapdh*) was used to normalize mRNA levels. Relative gene expression was analyzed with the 2^−ΔΔCt^ method [53]. The expression levels of various genes were expressed as fold-differences compared to in the sham group.

### 4.9. Implant Surgery

At 4 weeks after sham operation or OVX-surgery, 12-week-old female SD rats were anesthetized by inhalation with 1–2% isoflurane. Implants were placed only in the left femurs. A 1-mm diameter × 2-mm length implant site was prepared at 9 mm from the distal edge of the femur by drilling with a 0.8-mm round burr and was enlarged using reamers (#ISO 090 and 100) as described previously [49,51,55]. The total number of animals used was 32 (each group *n* = 8, implants *n* = 8), distributed among the sham-untreated, sham-UV, OVX-untreated, and OVX-UV implant groups (healing time 2 weeks).

### 4.10. Biomechanical Implant Push-in Test

The implant biomechanical push-in test was conducted to assess the biomechanical strength of bone–implant integration. The procedure details and method validation are described elsewhere [42,43]. After weeks 2 of healing, a testing machine (Instron 5544 electromechanical testing system; Instron, Norwood, MA, USA) equipped with a 2000 N load cell and custom-made pushing rod (diameter 0.8 mm) was used to load the implant vertically downward at a crosshead speed of 1 mm/min. The push-in value was determined by measuring the peak of the load–displacement curve, which was calibrated for the implant deviation angle.

### 4.11. Statistical Analysis

We used three samples for the cell culture studies and the number of implants for the implant push-in test was eight. Statistical analyses were carried out using statistics software (GraphPad Prism 6, GraphPad, Inc., San Diego, CA, USA). To evaluate the significance of differences between groups, two-way analysis of variance was performed. If necessary, a post hoc Bonferroni test was conducted to perform multiple comparison tests. Welch’s *t*-test was also used to compare the two groups. All data are expressed as the group mean ± standard deviation. A probability level of 0.05 or less was accepted as significant.

## 5. Conclusions

Our data show that UV-photofunctionalization enhanced rapid mechanical anchorage under post-menopausal osteoporosis conditions. Based on these results, UV-photofunctionalization is a promising option for inducing high levels of mechanical anchorage even under osteoporotic conditions and may contribute to improvements in current implant therapies in the dental and orthopedic fields.

## Abbreviations

ALP	Alkaline phosphatase
BMP-2	Bone morphogenetic protein-2
BrdU	5-bromo-2′ deoxyuridine
cDNA	Complementary DNA
Col1a1	collagen type I alpha 1 chain
ddH_2_O	double-distilled H_2_O
Gapdh	Glyceraldehyde-3-phosphate dehydrogenase
OVX	Ovariectomy
PCR	Polymerase chain reaction
WST	Water-soluble tetrazolium salts
